# Strength together: examining risk and protective factors associated with dementia and cognitive impairment in Aboriginal and Torres Strait Islander peoples through harmonisation of landmark studies

**DOI:** 10.1186/s12883-024-03688-y

**Published:** 2024-06-01

**Authors:** Huong X. T. Nguyen, Zoë Hyde, Bridgette J. McNamara, Jo-anne Hughson, Kylie Radford, Sarah Russell, Leon Flicker, Rachel Quigley, Roslyn Malay, Edward Strivens, Adrienne Withall, Louise Lavrencic, Brian Draper, Kim Delbaere, Robert Cumming, Dina LoGiudice

**Affiliations:** 1 Department of Medicine - Royal Melbourne Hospital, The University of Melbourne, Royal Park Campus, Administration Building 21, 34 -54 Poplar Road, Melbourne, VIC 3050 Australia; 2 Western Australian Centre for Health and Ageing, Medical School, University of Western Australia, Perth, Australia; 3 Centre for Aboriginal Medical and Dental Health, Medical School, University of Western Australia, Perth, Australia; 4 Centre for Epidemiology and Biostatistics, University of Melbourne, Melbourne, VIC Australia; 5 Barwon South-West Public Health Unit, Barwon Health, Geelong, VIC Australia; 6 Neuroscience Research Australia, Sydney, Australia; 7 School of Psychology, University of New South Wales, Sydney, Australia; 8 Ageing Futures Institute, University of New South Wales, Sydney, Australia; 9 College of Medicine and Dentistry, James Cook University, Cairns, QLD Australia; 10 Cairns and Hinterland Hospital and Health Service, Cairns, QLD Australia; 11 School of Population Health, University of New South Wales, Sydney, Australia; 12 Falls, Balance and Injury Research Centre, Neuroscience Research Australia, Sydney, Australia; 13 Discipline of Psychiatry and Mental Health, University of New South Wales, Sydney, Australia; 14 School of Public Health, University of Sydney, Sydney, Australia

**Keywords:** Ageing, Indigenous health, Epidemiology, Cohort studies, Preventative health

## Abstract

**Background:**

Rates of dementia for Aboriginal and Torres Strait Islander peoples are three to five times greater compared to non-Indigenous Australians, with earlier age of onset. However, the risk and protective factors that drive these higher rates vary across existing cohort studies, with minimal findings on the role of vascular risk factors beyond stroke. Harmonisation of data across studies may offer greater insights through enhanced diversity and strengthened statistical capabilities. This study aims to combine three landmark cohort studies of Aboriginal and Torres Strait Islander participants to better understand the determinants of cognitive health and dementia.

**Methods/design:**

Three cohort studies - the Kimberley Healthy Adults Project (KHAP, *N* = 363), Koori Growing Old Well Study (KGOWS, *N* = 336) and Torres Strait Dementia Prevalence Study (TSDPS, *N* = 274) - share a similar research methodology with demographic, medical history, psychosocial factors, cognitive tests and consensus clinical diagnoses of cognitive impairment and dementia. Associations between risk and protective factors of interest and the presence of dementia and/or cognitive impairment diagnoses will be evaluated by univariable and multivariable logistic regression in a harmonised cross-sectional cohort of 898 participants. Factors associated with incident dementia and/or cognitive impairment will be assessed in a subset of KHAP (*n* = 189) and KGOWS participants (*n* = 165) who were available in longitudinal follow-up, after exclusion of those with baseline dementia or cognitive impairment. Analyses in relation to outcome measure of death or dementia will be conducted to account for the competing risk of death. Logistic regression will be used to evaluate the association between the individual components of the 16-component Kimberley Indigenous Cognitive Assessment (KICA) tool and the presence of dementia and cognitive impairment determined by independent consensus diagnoses. Multivariable binary logistic regression will be used to adjust for the effect of confounding variables. Results will be reported as odds ratios (OR) with 95% confidence intervals (95% CI).

**Discussion:**

Greater understanding of risk and protective factors of dementia and cognitive impairment relevant to Aboriginal and Torres Strait Islander peoples may improve approaches across the life course to delay cognitive decline and reduce dementia risk.

**Supplementary Information:**

The online version contains supplementary material available at 10.1186/s12883-024-03688-y.

## Background

Survivorship among Aboriginal and Torres Strait Islander peoples is increasing, with many living long and healthy lives. At the same time, rates of cognitive impairment and dementia for Aboriginal and Torres Strait Islander peoples are three to five times greater compared to non-Indigenous Australians, with earlier age of onset [1–3]. Aboriginal and Torres Strait Islander people’s health and wellbeing is multifaceted and shaped by historical, cultural, political and socioeconomic dimensions. The legacy of colonisation and austere government policies have ongoing repercussions for many older Aboriginal and Torres Strait Islander peoples, some (estimated 1 in 7 over the age of 50) of whom are from the Stolen Generation - children forcibly removed from their families and raised in institutions, foster homes or adopted by non-Indigenous families between 1910s and 1970s [5]. Non-communicable and chronic diseases contribute much to the disparities in life expectancy between Aboriginal and Torres Strait Islander peoples and non-Indigenous Australians, which has only decreased marginally over the last decade from approximately 10 to 8 years [6, 7]. More is needed to promote brain health and ageing in this unique population over the life-course.

Three landmark Australian studies involving Aboriginal and Torres Strait Islander cohorts from Western Australia (WA), New South Wales (NSW), and the Torres Strait in Far North Queensland have researched factors associated with dementia. In a cross-sectional study of 363 Aboriginal people aged 45 years and older living in the remote Kimberley region of WA, dementia was associated with smoking, stroke, epilepsy, head trauma, poor mobility, incontinence and falls, after adjustment for age, sex and education. On follow-up, only age and head trauma endured as longitudinal risk factors for cognitive decline [8, 9]. In a cohort of 336 participants in the major city of Sydney and regional NSW, multivariable analysis highlighted age, childhood trauma, unskilled work, stroke and head injury as independent predictors of all-cause dementia alongside comorbid factors such as depression, current high risk alcohol use, low Body Mass Index (BMI), mobility impairment, functional impairment, incontinence, hospitalization in the past year, residing in residential care, social isolation and low physical activity levels [2, 10]. Older age, male sex, unskilled work history, and polypharmacy were associated with incident dementia and Mild Cognitive Impairment (MCI) [11]. On follow-up, age, male sex, unskilled work, polypharmacy and Apolipoprotein E allele 4 (APOE ε4) were longitudinal risk factors for cognitive decline. However, similar to the Kimberley study, the competing risk of mortality was not taken into consideration, which likely impacted these longitudinal results [9]. In the Torres Strait, cerebrovascular disease, chronic kidney disease, and older age were significantly associated with dementia in a cohort of 274 participants [12]. Vascular risk factors other than stroke, such as diabetes and hypertension, were not associated with dementia in any of the cohorts, possibly due to the ubiquity of these conditions, non-linear risk relationships or limited statistical power. This is consistent with the findings of a recent systematic review that highlighted the limited data on risk factors for vascular dementia and a lack of geographical representation for many risk factors for dementia [13].

### Study aims

The aim of this research is to elucidate risk factors associated with cognitive impairment and dementia by combining landmark cohort studies involving Aboriginal and Torres Strait Islander participants. The Kimberley Healthy Adults Project (KHAP), Koori Growing Old Well Study (KGOWS) and the Torres Strait Dementia Prevalence Study (TSDPS) share similar study methodologies and outcome measures that allow for harmonisation to examine both risk and protective factors of brain health and impairment in a larger combined cohort that may offer greater insights through enhanced diversity and statistical capability.

Primary outcomes:

1. Elucidate risk and protective factors associated with dementia and/or cognitive impairment in a combined cross-sectional dataset of Aboriginal and Torres Strait Islander participants.
2. Elucidate risk and protective factors associated with incident dementia and/or cognitive impairment in a combined longitudinal dataset of Aboriginal and Torres Strait Islander participants.

Secondary outcomes:

1. Identify items in the Kimberley Indigenous Cognitive Assessment (KICA) or modified KICA (mKICA) that are most sensitive for dementia or cognitive impairment in the harmonised cross-sectional dataset.

## Methods/design

### Design

The KHAP study was undertaken from 2004 to 2006 and involved 363 Aboriginal participants aged 45 and older from different communities in the remote Kimberley region of WA [1]. The KGOW study recruited 336 participants aged 60 and older from five Aboriginal communities, two from metropolitan Sydney and three in regional coastal areas of NSW between 2010 and 2012 [14]. Follow-up of participants occurred after six and seven years for KGOWS-II and KHAP wave 2, respectively. The TSDP study was conducted between 2015 and 2018 and recruited 274 participants aged 45 and older from five mainland and 18 island communities across the Torres Strait and Northern Peninsula [3]. A summary of the studies and number of participants is provided in Table 1. All three cohort studies worked in partnership with local community organisations and Aboriginal and Torres Strait Islander members and researchers to support recruitment, data collection, and feedback to communities to ensure culturally safe engagement with participants. Risk factors were collected using a structured interview protocol and encompassed demographic, medical, and psychosocial factors collected from participants and family carers where available. This comprehensive assessment was independently evaluated by a panel of clinical specialists to establish clinical diagnoses by consensus. All three cohort studies used the KICA, a screening tool for older Aboriginal and Torres Strait Islander peoples, that was validated in the remote Kimberley region of WA [15], modified for urban and regional dwellers in the form of the mKICA [16] and adapted for the Torres Strait [17]. A shortened version (KICA-Screen) has been validated for face-to-face and telehealth administration [17, 18].

**Table 1:** Descriptive summary of landmark studies

Study/Cohort	Location	Age (years)	Sample Size (*N*=)	Participants (*n*=)
Kimberley Healthy Adults Project (KHAP)				
Wave 1 (W1) 2004–2006 [1, 8]	Remote WA	≥ 45	363	289 with normal cognition; 45 with dementia; 29 with CIND*.
Wave 2 (W2) 2011–2013 [9]	Remote WA	≥ 45	398‡	. Of 363 W1 participants, 184 completed all W2 assessments, 5 had cognitive assessment only, 109 had died and 65 declined to participate or could not be located. 105 new participants were recruited at W2. W2 cohort: 226 with normal cognition; 29 with dementia; 33 with CIND; 1 missing diagnosis; had 109 died.
Koori Growing Old Well Study (KGOWS)				
Wave 1 (W1) 2010–2012 [2, 14, 19]	Urban & Regional NSW	≥ 60	336	249 with normal cognition; 45 with dementia; 38 with MCI†; 4 with other cognitive disorder.
Wave 2 (W2) 2016–2018 [20]	Urban & Regional NSW	≥ 60	233‡	Of 336 W1 participants, 165 were followed-up, 68 had died and 103 did not participate. W2 cohort: 105 with normal cognition; 10 with dementia at baseline; 16 with incident dementia; 34 with MCI; 68 had died.
Torres Strait Dementia Prevalence Study (TSDPS)				
Wave 1 (W1) 2015–2018 [3, 12]	Torres Strait	≥ 45	274	175 with normal cognition; 39 dementia; 60 CIND.

CIND = Cognitive Impairment No Dementia; MCI = Mild Cognitive Impairment; NSW = New South Wales; WA = Western Australia

*CIND is defined as those with a decrement in cognitive functioning that met DSM-IV-TR criteria for a cognitive or amnestic disorder but without significant loss in activities of daily living (ADL) and not attributable to a psychiatric illness or intellectual disability

† MCI diagnoses in the KGOW cohort were reclassified as CIND based on DSM IV-TR diagnoses in line with the other cohorts for analyses

‡ Sample sizes include those who have died

### Research collaboration and partnerships

This project involves collaboration between different research teams across Australia. A Memorandum of Understanding (MOU) was agreed upon by chief investigators of the respective studies prior to the commencement of this project, according to cohort-specific governance processes, including Aboriginal and Torres Strait Islander community consultation and approval. The MOU specifies how data will be shared, stored, and handled between parties involved. It also details acknowledgments of researchers involved in the original cohort studies in any future publications or presentations. The Statistical Analysis Plan for this study was registered on the public platform Open Science Framework (https://osf.io/jv6sx/) prior to data sharing. Regular meetings with the research data leads will occur every two to three months to update our collaborators on progress of the study.

### Ethics

The KHAP, KGOW and TSDP studies all adhere to the Australian National Health and Medical Research Council (NHMRC) guidelines for ethical conduct for research involving Aboriginal and Torres Strait Islander People and communities. All studies have ethics approval from local and Aboriginal Health and Medical Research Councils. This project to harmonise these landmark studies has received ethics approval from the Aboriginal Health and Medical Research Council (AHMRC) 1362/18 & 1445/18; Western Australian Aboriginal Health Ethics Committee (WAAHEC) HREC no. 858; University of Melbourne Project ID 12,140 ; the Far North Queensland Human Research Ethics Committee HREC/2020/QCH/63,123–1432 and James Cook University H9079. The most important risk for this project involves participant confidentiality in the process of data sharing. Breach of confidentiality may lead to a lack of respect for research organisations and affect relationships with Aboriginal and Torres Strait Islander communities in the future. To ensure that this does not occur, we have specified in our ethics applications and MOU to share only de-identified data that will be stored on a secure, password-protected server, accessible only to research leads. This also means that any reporting or feedback of data and results will be in aggregate form without identification of individual participants. Our project governance structure with consultation from Indigenous governance groups also ensures that this research is in keeping with the values of the Aboriginal and Torres Strait participants and that outcomes are translated back to benefit community.

### Project governance

This research is currently embedded in the Let’s CHAT (Community Health Approaches To) Dementia in Aboriginal and Torres Strait Islander Communities project [21]. When Let’s CHAT comes to completion, governance will be transferred and provided under the auspices of the recently established On TRACK (Teaching, Research And Community Knowledges) national research program. On TRACK is a NHMRC-funded Centre for Research Excellence (CRE) collaboration, which aims to promote brain health and optimise detection of dementia and cognitive impairment in older Aboriginal and Torres Strait Islander peoples through engagement with Aboriginal primary health care services and adheres to the key principles for ethical conduct in research with Aboriginal and Torres Strait Islander peoples based on NHMRC and AHMRC guidelines [22, 23].

The harmonisation of existing cohort data does not directly involve partnership with Aboriginal Community Controlled Health Services. Aboriginal and Torres Strait Islander governance is achieved through an Indigenous Reference Group (IRG) whose members are involved through the course of the project and are “representative of the group being studied and have knowledge or experience of the research matter” [22]. In its conceptualisation, this project has the support of the Let’s CHAT Dementia IRG and the KGOWS Steering Committee. The Let’s CHAT IRG comprises Elders and research team members with links to communities in Western Australia and the Torres Strait. The KGOWS Steering Committee is led by Aboriginal researchers who were involved in the KGOW studies, in conjunction with representatives from each of the partnering Aboriginal communities and independent Aboriginal and Torres Strait Islander members with expertise in brain health and ageing. The TSDPS was also governed by an Indigenous Knowledge Circle with members continuing to provide oversight of ongoing Torres Strait studies conducted by the Healthy Ageing Research Team (HART). Many members of the Let’s CHAT IRG have elected to continue as members of the On TRACK IRG and will provide ongoing governance for this project. This group will comprise of at least six Aboriginal and Torres Strait Islander members who will meet three times a year. In addition to annual project progress reports submitted to the ethic committees, presentations to the IRG will occur during these meetings. This will ensure that Indigenous governance groups are involved in interpretation of the research results and plans for knowledge translation. The On TRACK CRE will also increase Indigenous research capacity through training and employment of Indigenous Research Officers who will be involved in data collection, research analysis, publication writing and dissemination of research findings back to community and the wider sector. Indigenous Research Officers and IRG members will be co-authors in peer-reviewed publications and, where possible, co-presenters at national and international conferences. This will ensure cultural safety and the prioritisation of Indigenous values and perspectives in interpretation of study findings and their translation into clinical practice.

### Data for harmonisation

#### Dementia diagnosis

Diagnoses in each of the studies were based on data obtained through comprehensive medical assessment inclusive of medical, behavioural, neurological, cognitive and collateral caregiver measures performed by a trained specialist (geriatrician, older person psychiatrists, general physician) in dementia assessment. De-identified data from clinical assessments were reviewed by a panel of specialists to obtain consensus diagnoses based on the Diagnostic and Statistical Manual for Mental Disorders, 4th Edition (DSM IV-TR) [24]. Dementia subtypes of Alzheimer’s, vascular, mixed, or other were specified where possible. For the purpose of analyses, participants were reclassified based on DSM IV-TR diagnoses as having normal cognition, dementia, MCI [2] or CIND [8, 12]. The CIND and MCI groups demonstrated a decrement in cognitive functioning that met DSM-IV-TR criteria for a cognitive or amnestic disorder that was not attributable to a psychiatric illness or intellectual disability, but without significant loss in activities of daily living (ADL). For the harmonised dataset, MCI diagnoses in the KGOWS cohort were reclassified as CIND based on DSM IV-TR diagnoses in line with the other cohorts for analyses. Dementia subtypes of Alzheimer’s, vascular, mixed, or other were specified where possible based on the DSM IV-TR.

#### Variables of interest

Risk and protective variables of interest associated with dementia are derived from the 2020 Lancet report, the World Health Organisation (WHO) guidelines, Cardiovascular Risk Factors Aging, and Incidence of Dementia (CAIDE) index, and Australian National University Alzheimer’s Disease Risk Index (ANU-ADRI) and summarised in Table 2. These are life-course risk and protective factors for dementia in the general population and well established in the literature [13, 25–27]. We have included these measures for analysis across the three studies to establish whether the same risk factors are relevant to Aboriginal and Torres Strait peoples. Studies have also shown that certain syndromes such as falls, executive dysfunction, depressive symptoms and urinary incontinence may be more common in older people with cerebral white matter changes suggestive of cognitive vulnerability and impairment [28, 29]. Measures of mobility impairment, falls, sleep disturbance, urinary incontinence, and impairments in ADLs will be included to look at these syndromes. Additionally, history of cardiovascular, renal, or cerebrovascular disease inclusive of stroke or a transient ischaemic attack are included as markers of vascular comorbid conditions.

**Table 2:** Compilation of risk factors of interest based on existing literature

Measure	Data available
Less education (< 10 years) *†‡§	Simple measure in KHAP W1. Levels of education available in KHAP W2, KGOWS and TSDPS.
Hearing impairment*	Yes
Physical inactivity/exercise *†§	Simple measure available in KGOWS but not in KHAP or TSDPS
Cognitive stimulation †§	Skilled work/jobs as a proxy measure in KGOWS only
Head injury with loss of consciousness *§	Yes
Diabetes †*§	Yes
Hyperlipidaemia/ high cholesterol †‡	KHAP W2, KGOWS and TSDPS only
Hypertension *†‡	Yes
Infrequent social contact/social isolation *†§	Only available from KGOWS data Subjective loneliness, living alone, social activity participation in prior 3 months for 10 activities
Diet (e.g. fish intake) †§	No
Alcohol consumption *†§	Yes
Air pollution *	No
Smoking *†§	Yes
Obesity (Body mass index > 30) †*‡	Only in KHAP W2 and KGOWS
Depression/Depressive symptoms *†§	Yes
Older Age ‡§	Yes
Sex §	Yes

* Dementia prevention, intervention, and care: 2020 report of the *Lancet* Commission [26]

†World Health Organisation (WHO) guidelines [30]

‡ Cardiovascular Risk Factors Aging, and Incidence of Dementia (CAIDE) Risk Score [31]

§ Australian National University Alzheimer’s Disease Risk Index (ANU-ADRI) [32]

Note: alcohol consumption is a protective factor in the ANU-ADRI but excessive alcohol consumption (> 21 units per week) is a risk factor in the Lancet 2020 Commission Report and in the WHO guidelines

#### Data harmonisation

Data will be harmonised using the Stata statistical package (StataCorp, College Station, Texas), version 17. Variables of interest will be recoded to be consistent across datasets prior to combination into a single final dataset. Data harmonisation utilises the best available information. This means that patient self-reports for a given variable may be corroborated by information from family reports, medications, primary care software derived medical history and medications where available. For instance, the variable diabetes combines self-reports of the condition (yes/no), family informant reports of the condition (yes/no), and medication history (diabetic medication by self-reports or in medication review). Medication lists available for the participants will be coded using the World Health Organization’s Anatomical Therapeutic Chemical (ATC) Classification and allow for sub-analyses of outcomes in relation to medication categories of interest such as anticholinergics, analgesics, anti-psychotics and so forth [33]. The full details of variables of interest in the pooled cross-sectional dataset are provided in the appendices (Appendix 1.1).

Based on the variables of interest available in each dataset (Table 2), KHAP wave 2, KGOWS-I and TSDPS will be harmonised in cross-section, with a combined cohort of 898 Aboriginal and Torres Strait Islander participants, 244 (27%) of whom will have a diagnosis of dementia or CIND/MCI. For longitudinal analyses, data from KHAP wave 1 will be harmonised with KGOWS-I and combined with diagnoses at baseline (wave 1) and follow-up (wave 2) for analysis of factors of interest in a smaller subset of 354 participants as summarised in the appendices (Appendix 1.2). There is no longitudinal data available for TSDPS at the present time.

#### Missing data and outliers

Data harmonisation utilises the best available information to minimise missing data. In circumstances where missing data is substantial (> 5% for a single variable), imputations may be considered for predictor variables and covariates.

#### Data analyses

Data will be analysed using the Stata statistical package (StataCorp, College Station, Texas), version 17. Analyses will be performed on harmonised cross-sectional and longitudinal data. Associations between risk and protective factors of interest and the presence of dementia and/or cognitive impairment based on consensus diagnoses will be evaluated by univariable and multivariable binary logistic regression. Analyses by dementia subtype will also be conducted to ascertain risk factors of importance between groups. In the longitudinal cohort, risk factors of interest will be determined from baseline measures (W1), compared with consensus diagnoses at follow-up (wave 2) and analysed in relation to outcome measures of incident dementia and/or cognitive impairment using univariable and multivariable logistic regression. To account for the competing interest of death (*n* = 177 before wave 2 follow-up), multinominal logistic regression will be used to estimate the association between variables of interest and the following outcomes: (1) neither cognitive impairment nor dementia nor died; (2) cognitive impairment or dementia during the study; and (3) death between baseline and follow-up. Results will be reported as odds ratios (OR) with 95% confidence intervals (95% CI). Multivariable binary logistic regression will be used to adjust for the effect of confounding variables that are anticipated to be associated with dementia such as age, sex, education and study cohort. Variables that are significant in univariable analyses will be entered into a multivariable model, after which a process of clinical judgement and examination of measures including the Akaike and Bayesian information criteria will be employed to decide which variables to retain in the final multivariable model.

#### Analyses of secondary outcome

Logistic regression will be used to evaluate the association between the individual components of the 16-question KICA, in its original and modified forms, and the presence of CIND and dementia diagnoses. Results will be reported as OR with 95% CI. This will allow for the identification of parameters in the KICA that are most sensitive for dementia. Sensitivity and specificity values and optimal cut-off scores will be informed by formulation of receiver operating characteristic (ROC) curves and area under the curve (AUC) calculations. Multivariable logistic regression will be used to adjust for the effect of potential confounding variables such as age, sex, education and study cohort. A resulting prototype short scale will then be validated in a separate cohort of Aboriginal and Torres Strait Islander peoples.

## Discussion

### Strengths and weaknesses

The pooled dataset brings together three Australian landmark cohort studies of dementia involving Aboriginal and Torres Strait Islander peoples with the potential to offer greater insights through enhanced statistical power. Risk and protective factors associated with dementia can be determined from the combined cross-sectional cohort of 898 participants but causality cannot be inferred. The pooled longitudinal cohort is more modest in size and contains more coarse measures of variables of interest, however, it will allow for analyses of incident dementia and cognitive impairment. We are also limited by the risk factor data available across all three cohorts. Some important omissions include physical inactivity, cognitive stimulation, social isolation, diet and adverse childhood experiences. There are also many social and cultural determinants of health which are yet to be measured and investigated in relation to dementia risk, which are likely to be important for optimising brain health and reducing dementia risk.

### Anticipated research and implications for this harmonised dataset

The 2020 Lancet Commission on Dementia Prevention, Intervention and Care suggested that at a population level, up to 40% of dementia may theoretically be preventable by eliminating certain risk factors [26]. These included less education in early life, hypertension, hearing impairment, smoking, obesity, depression, physical inactivity, diabetes, social isolation, traumatic brain injury, excessive alcohol consumption and air pollution as key risk factors in the development of dementia. Studies suggest that the preventable risk of dementia is greater in some Indigenous populations than global estimates, though risk profiles may differ between Indigenous groups [34–36] and when compared to other non-Indigenous ethnic groups [35, 37]. Greater understanding of risk and protective factors of dementia relevant to Aboriginal and Torres Strait Islander peoples may assist in preventative efforts to promote brain health and resilience over the life-course.

Risk evaluation tools are useful in primary care to identify high-risk patients and provide a framework for managing risk factors associated with chronic diseases. This has been successfully demonstrated in several fields of medicine such as cardiovascular disease (e.g. CHADs-VASC; Framingham cardiovascular risk score) and osteoporosis (FRAX; Garvan Institute Fracture Risk Calculator) [38–41]. The risk and protective factors identified from the harmonised data may allow for the development of a strength-based tool for assessment of brain health for older Aboriginal and Torres Strait Islander peoples. We envisage that this inexpensive tool would be linked to Best Practice Guidelines to provide a framework for patient education, optimise the management of conditions impacting on brain health and enhance appropriate follow-up at the primary care interface. Through the On TRACK CRE, we will translate this research with Indigenous engagement to ensure community informed and culturally safe care.

### Final conclusions

Greater understanding of risk and protective factors for dementia relevant to Aboriginal and Torres Strait Islander peoples may improve public health approaches to promote brain health across the life course. It could also improve timely and supported diagnosis of dementia and enables early interventions, minimisation of harm through de-prescribing of medications, review of comorbid conditions and discussion of future planning in culturally meaningful ways. This research aligns with community efforts to ‘Close the Gap’ and reduce disparities in rates of dementia and outcomes between Indigenous and non-Indigenous Australians.

### Electronic supplementary material

Below is the link to the electronic supplementary material.

Supplementary Material 1

## Data Availability

Data will not be made publicly available due to ethical requirements. External collaborators can apply to the KHAP, KGOWS and HART steering committees for further information about individual datasets for future studies.

## Declarations

The authors report no disclosures relevant to the manuscript. This study has received ethics approval from the Aboriginal Health and Medical Research Council (AHMRC), Western Australian Aboriginal Health Ethics Committee (WAAHEC), University of Melbourne and Far North Queensland and James Cook University Human Research Ethics Committees. Informed consent was obtained from all subjects and/or their legal guardians informed in the original research. This research is carried out in accordance with the key principles for ethical conduct in research with Aboriginal and Torres Strait Islander peoples based on NHMRC and AHMRC guidelines.

## Abbreviations

ADL: Activities of Daily Living
AHMRC: Aboriginal Health and Medical Research Council
ATC: Anatomical Therapeutic Chemical
ANU: ADRI-Australian National University Alzheimer’s Disease Risk Index
APOE ε4: Apolipoprotein E allele 4
AUC: Area Under the Curve
AUDIT: C-Alcohol use disorders identification test for consumption
BMI: Body Mass Index
CAIDE: Cardiovascular Risk Factors Aging, and Incidence of Dementia
DSM: IV-Diagnostic and Statistical Manual-IV
CIND: Cognitive Impairment No Dementia
DSM IV: TR-Diagnostic and Statistical Manual for Mental Disorders, 4th Edition
EFST: Elderly Falls Screening Test
HART: Healthy Ageing Research Team
IRG: Indigenous Reference Group
KDS: Kilsyth Disability Scale
KHAP: Kimberley Healthy Adults Project
KICA: Kimberley Indigenous Cognitive Assessment
KICA: ADL-Kimberley Indigenous Cognitive Assessment-Activities of Daily Living
KICA: Dep-Kimberley Indigenous Cognitive Assessment-Depression Scale
KGOWS: Koori Growing Old Well Study
Let’s CHAT Dementia: Let’s CHAT (Community Health Approaches To) Dementia
mKICA: modified Kimberley Indigenous Cognitive Assessment
MCI: Mild Cognitive Impairment
MMSE: Mini-Mental State Examination
NHMRC: National Health and Medical Research Council
NSW: New South Wales
On TRACK: On TRACK (Teaching, Research And Community Knowledges)
OSF: Open Science Framework
OR: Odds ratios
ROC: Receiver Operating Characteristic
TSDPS: Torres Strait Dementia Prevalence Study
WA: Western Australia
WAAHEC: Western Australian Aboriginal Health Ethics Committee
WHO: World Health Organisation

## References

[1] Smith K, Flicker L, Lautenschlager NT, Almeida OP, Atkinson D, Dwyer A (2008). High prevalence of dementia and cognitive impairment in indigenous australians. Neurology.

[2] Radford K, Lavrencic LM, Delbaere K, Draper B, Cumming R, Daylight G (2019). Factors Associated with the high prevalence of dementia in older Aboriginal australians. J Alzheimers Dis.

[3] Russell SG, Quigley R, Thompson F, Sagigi B, LoGiudice D, Smith K (2021). Prevalence of dementia in the Torres Strait. Australas J Ageing.

[4] Prince M, Bryce R, Albanese E, Wimo A, Ribeiro W, Ferri CP (2013). The global prevalence of dementia: a systematic review and metaanalysis. Alzheimers Dement.

[5] Health, AIo. Welfare. Aboriginal and Torres Strait Islander Stolen generations aged 50 and over. Australian Institute of Health and Welfare; 2018.

[6] Aboriginal. and Torres Strait Islander life expectancy 2020–2022 [press release]. Canberra, 2020–2022.

[7] Collins HP, AM Q, Kalisch D. Life expectancy and mortality of Aboriginal and Torres Strait Islander people.

[8] Smith K, Flicker L, Dwyer A, Atkinson D, Almeida OP, Lautenschlager NT (2010). Factors associated with dementia in Aboriginal australians. Australian New Z J Psychiatry.

[9] Lo Giudice D, Smith K, Fenner S, Hyde Z, Atkinson D, Skeaf L (2016). Incidence and predictors of cognitive impairment and dementia in Aboriginal australians: a follow-up study of 5 years. Alzheimer’s Dement J Alzheimer’s Assoc.

[10] Radford K, Delbaere K, Draper B, Mack HA, Daylight G, Cumming R (2017). Childhood Stress and Adversity is Associated with late-life dementia in Aboriginal australians. Am J Geriatr Psychiatry.

[11] Lavrencic LM, Delbaere K, Broe GA, Daylight G, Draper B, Cumming RG (2022). Dementia incidence, APOE genotype, and risk factors for Cognitive decline in Aboriginal australians: a longitudinal cohort study. Neurology.

[12] Russell SG, Quigley R, Thompson F, Sagigi B, Miller G, LoGiudice D (2022). Factors associated with the increased risk of dementia found in the Torres Strait. Australas J Ageing.

[13] Anstey KJ, Ee N, Eramudugolla R, Jagger C, Peters R (2019). A systematic review of Meta-analyses that evaluate risk factors for dementia to evaluate the quantity, Quality, and global representativeness of evidence. J Alzheimers Dis.

[14] Radford K, Mack HA, Robertson H, Draper B, Chalkley S, Daylight G (2014). The Koori growing Old Well Study: investigating aging and dementia in urban Aboriginal australians. Int Psychogeriatr.

[15] LoGiudice D, Smith K, Thomas J, Lautenschlager NT, Almeida OP, Atkinson D (2006). Kimberley Indigenous Cognitive Assessment tool (KICA): development of a cognitive assessment tool for older indigenous australians. Int Psychogeriatr.

[16] Jackson Pulver L, Broe A, Grayson D, Chalkley S, Flicker L, Daylight G et al. Dementia screening for urban Aboriginal Australians: The modified kimberly indigenous cognitive assessment (mKICA). 2012.

[17] LoGiudice D, Strivens E, Smith K, Stevenson M, Atkinson D, Dwyer A (2011). The KICA screen: the psychometric properties of a shortened version of the KICA (Kimberley Indigenous Cognitive Assessment). Australas J Ageing.

[18] Russell S, Quigley R, Strivens E, Miller G, Norrie J, Craig D (2021). Validation of the Kimberley Indigenous Cognitive Assessment short form (KICA-screen) for telehealth. J Telemed Telecare.

[19] Radford K, Mack HA, Draper B, Chalkley S, Daylight G, Cumming R (2015). Prevalence of dementia in urban and regional Aboriginal australians. Alzheimer’s Dement J Alzheimer’s Assoc.

[20] Lavrencic LM, Delbaere K, Broe GA, Daylight G, Draper B, Cumming RG (2022). Dementia incidence, APOE genotype, and risk factors for Cognitive decline in Aboriginal australians: a longitudinal cohort study. Neurology.

[21] Bradley K, Smith R, Hughson J-A, Atkinson D, Bessarab D, Flicker L et al. Let’s CHAT (community health approaches to) dementia in Aboriginal and Torres Strait Islander communities: protocol for a stepped wedge cluster randomised controlled trial. BMC Health Serv Res. 2020;20(1). 10.1186/s12913-020-4985-1 PMC7069169 32164678

[22] Council AHaMR. AH&MRC Ethical Guidelines Key Principles (2020) V2.0. 2020.

[23] Council NHaMR. Keeping research on track II: a companion document to ethical conduct in research with Aboriginal and Torres Strait Islander Peoples and communities: guidelines for researchers and stakeholders. Canberra National Health and Medical Research Council; 2018.

[24] Association AP (1997). Diagnostic and statistical manual of mental disorders.

[25] Livingston G, Sommerlad A, Orgeta V, Costafreda SG, Huntley J, Ames D (2017). Dementia prevention, intervention, and care. Lancet.

[26] Livingston G, Huntley J, Sommerlad A, Ames D, Ballard C, Banerjee S (2020). Dementia prevention, intervention, and care: 2020 report of the Lancet Commission. Lancet.

[27] Peters R, Booth A, Rockwood K, Peters J, D’Este C, Anstey KJ (2019). Combining modifiable risk factors and risk of dementia: a systematic review and meta-analysis. BMJ open.

[28] Alagiakrishnan K, Hsueh J, Zhang E, Khan K, Senthilselvan A (2013). White matter disease severity of the brain and its association with geriatric syndromes. Postgrad Med.

[29] Sakakibara R, Panicker J, Fowler CJ, Tateno F, Kishi M, Tsuyuzaki Y (2012). Vascular incontinence: incontinence in the elderly due to ischemic white matter changes. Neurol Int.

[30] World Health Organisation. Risk reduction of cognitive decline and dementia: WHO guidelines. 2019. 31219687

[31] Kivipelto M, Ngandu T, Laatikainen T, Winblad B, Soininen H, Tuomilehto J (2006). Risk score for the prediction of dementia risk in 20 years among middle aged people: a longitudinal, population-based study. Lancet Neurol.

[32] Anstey KJ, Cherbuin N, Herath PM, Qiu C, Kuller LH, Lopez OL (2014). A self-report risk index to predict occurrence of dementia in three independent cohorts of older adults: the ANU-ADRI. PLoS ONE.

[33] Organization WH. Guidelines for ATC classification and DDD assignment. 1996.

[34] MacDonald JP, Barnes DE, Middleton LE (2015). Implications of risk factors for Alzheimer’s disease in Canada’s indigenous population. Can Geriatr J.

[35] Ma’u E, Cullum S, Cheung G, Livingston G, Mukadam N (2021). Differences in the potential for dementia prevention between major ethnic groups within one country: a cross sectional analysis of population attributable fraction of potentially modifiable risk factors in New Zealand. Lancet Reg Health - Western Pac.

[36] Thompson F, Russell S, Quigley R, Sagigi B, Taylor S, McDonald M (2022). Potentially preventable dementia in a First Nations population in the Torres Strait and Northern Peninsula Area of North Queensland, Australia: a cross sectional analysis using population attributable fractions. Lancet Reg Health West Pac.

[37] Ashby-Mitchell K, Burns R, Shaw J, Anstey K (2017). Proportion of Dementia in Australia explained by common modifiable risk factors. Alzheimer’s Res Therapy.

[38] Zhang J, Lenarczyk R, Marin F, Malaczynska-Rajpold K, Kosiuk J, Doehner W (2021). The interpretation of CHA2DS2-VASc score components in clinical practice: a joint survey by the European Heart Rhythm Association (EHRA) Scientific Initiatives Committee, the EHRA Young Electrophysiologists, the association of cardiovascular nursing and allied professionals, and the European Society of Cardiology Council on Stroke. EP Europace.

[39] Schnabel RB, Sullivan LM, Levy D, Pencina MJ, Massaro JM, D’Agostino RB (2009). Development of a risk score for atrial fibrillation (Framingham Heart Study): a community-based cohort study. Lancet.

[40] Kanis J, McCloskey E, Johansson H, Oden A, Ström O, Borgström F (2010). Development and use of FRAX® in osteoporosis. Osteoporos Int.

[41] Nguyen ND, Frost SA, Center J, Eisman JA, Nguyen TV (2008). Development of prognostic nomograms for individualizing 5-year and 10-year fracture risks. Osteoporos Int.

