# Supplementary material for: Strength together: examining risk and protective factors associated with dementia and cognitive impairment in Aboriginal and Torres Strait Islander peoples through harmonisation of landmark studies
Source: BMC Neurol. 2024 Jun 1;24:185. doi: 10.1186/s12883-024-03688-y (PMC11143581; doi:10.1186/s12883-024-03688-y)
Supplement: Supplementary file 1 — Supplementary Material 1 [file 12883_2024_3688_MOESM1_ESM.docx]

**Supplementary Information**

**Appendix 1.1: Harmonised cross-sectional dataset and variables of interest**

| **Variables** | **KHAP W2** | **KGOWS-I** | **TSDPS** | **COMBINED** |
| --- | --- | --- | --- | --- |
| 1. Age | Continuous  Categorical as 5-year bracket | Years (continuous) | Continuous  Categorical as 5-year bracket | **age**  Continuous |
| 1. Sex | Female/male | Female/male | Female/male | **sex**  1=male;  2=female |
| 1. Locality | All from the Kimberley (remote) | Urban/regional | All from the Torres Strait (remote) | **locality**  1=urban; 2=regional; 3=remote |
| 1. Education attainment | Self-reports: Went to school no/yes  Primary no/yes.  years continuous  Secondary no/yes; years continuous  Any further study no/yes=  Years (continuous) | Years (continuous) | Primary no/yes Grade 8-10 no/yes  Grade 11-12 no/yes  Post school no/yes | **education**  0=no school; 1=primary school; 2=secondary school;  3=further education |
| 1. Smoking | Current smoking: no/yes  Previous smoking: no/yes | Self-reports: Current smoking  no/yes  Previous smoking no/yes | current smoking: no/yes  Previous smoking: no/yes | **smoking**  0= never smoked; 1= ex-smoker;  2= current smoker |
| 1. Alcohol | Current alcohol use yes/no; previous alcohol use yes/no  Drink when younger no/yes  Frequency: Drank most days/got drunk  Amount: how often get drink only sometimes vs.1-3 days/wk vs. 4-6 days/week vs. every day  Age started drinking: 4 categorical age brackets  Frequency: only sometimes vs. 1-3 days/wk vs. 4-6 days/week vs. every day  Quantity: 1-3 drinks 4-6 drinks/ until drunk | AUDIT-C: Current alcohol Abstinent/low risk/ high risk;  Past alcohol Abstinent/low risk/ high risk | Current alcohol use yes/no; previous alcohol use yes/no  Drink when younger no/yes  Frequency: Drank most days/got drunk  Amount: how often get drink only sometimes vs.1-3 days/wk vs. 4-6 days/week vs. every day  Age started drinking: 4 categorical age brackets  Frequency: only sometimes vs. 1-3 days/wk vs. 4-6 days/week vs. every day  Quantity: 1-3 drinks 4-6 drinks/ until drunk | **alcohol**  0= no alcohol;  1= ex-alcohol;  2= current alcohol  **Alcohol current intensity**  0=abstinent  1=low risk/only sometimes/1-3 days/wk/4-6 days/wk and 1-3 drinks;  2=high risk/everyday/4-6 drinks and 4-6 drinks a day/until drunk  **Alcohol past intensity**  0=abstinent;  1=low risk/only sometimes/1-3 days/wk/4-6 days/wk and 1-3 drinks;  2=high risk/everyday/4-6 drinks and 4-6 drinks a day/until drunk |
| 1. Diabetes | self-report: no/yes  family report: no/yes  mmex_diabetes: no/yes  diabetic medication: no/yes | self/family/carer report or medical assessment data: no/yes  medications* | self-report: no/yes  medications*  no/yes | **diabetes**  0=no;  1=yes |
| 1. Hypertension | self-report: no/yes  family report: no/yes  mmex_htn*: no/yes  SBP/DBP | self/family/carer report or medical assessment data: no/yes  SBP/DBP/MAP | self-report: no/yes | **hypertension**  0=no;  1=yes |
| 1. Hypercholesterolaemia/   Hyperlipidaemia/ dyslipidaemia | mmex_dyslipidaemia*: no/yes  drug_lipidlowering~: no/yes | self/family/carer report or medical assessment data: no/yes  medications* | no/yes  medications* | **hyperlipidaemia**  0=no;  1=yes |
| 1. Obesity | BMI calculated from height and w measures (continuous)  waist to hip ratio (continuous) | BMI calculated from height and weight measures (continuous)  waist to hip ratio (continuous) | Not consistently recorded.  Only available in past medical history notes for some with obesity as a diagnosis | **obesity**  BMI ≥ 30 or waist to hip ratio ≥ 1 or diagnosis of obesity  0=no;  1=yes |
| 1. Renal disease | self-report: no/yes  family report: no/yes  mmex_renal*: no/yes | self/family/carer report or medical assessment data: no/yes | self-report: no/yes | **renal**  0=no;  1=yes |
| 1. Cardiovascular disease | self-report: no/yes  family report: no/yes  mmex_ihd*: no/yes  mmex_ccf*: no/yes | self/family/carer report or medical assessment data: no/yes | self-report: no/yes | **cardiac**  0=no;  1=yes |
| 1. Cerebral vascular disease/stroke | self-report: no/yes  family report: no/yes  mmex_stroke*: no/yes | self/family/carer report or medical assessment data: no/yes | self-report: no/yes | **stroke**  0=no;  1=yes |
| 1. Epilepsy/seizures | mmex_epilepsy*: no/yes | self/family/carer report or medical assessment data: no/yes | self-report: no/yes | **epilepsy**  0=no;  1=yes |
| 1. Hearing problems | self-report: ears good no/yes/don’t know (NB. item reversed | self/family/carer report or medical assessment data: no/mild vs. moderate/severe | self report: ears good no/yes/don’t know (NB. item reversed) | **hearing**  0=no/mild; 1=yes/moderate/severe |
| 1. Vision problems | self-report: eyes good no/yes/don’t know (NB. item reversed | self/family/carer report or medical assessment data: no/mild vs. moderate/severe | self report: eyes good no/yes/don’t know (NB. item reversed | **vision**  0=no/mild; 1=yes/moderate/severe |
| 1. Head injury with loss of consciousness | self-report: no/yes  family report: no/yes  mmex_headinjury*: no/yes | self/family/carer report or medical assessment data : no/yes | self-report: no/yes | **head_injury**  0=no;  1=yes |
| 1. Depression | self-report: Feel down, sad or no good: no/yes  drug_antidepressant~: no/yes  mmex_antidepressant*: no/yes  KICA-Dep score (continuous) | self/family/carer report or medical assessment data: history of depression no/yes  mPHQ9 score (continuous) | self-report: Feel down, sad or no good: no/yes  KICA-Dep Score | **depression**  0=mPHQ9 < 9 or KICA-Dep < 8 or no on personal or informant history;  1=mPHQ9 ≥ 9 or KICA-Dep ≥8 or yes on personal or informant history  **depression score** Continuous |
| 1. Sleep disturbance | From depression questionnaire: sleeping not enough or too much no/yes | From depression questionnaire; self/family/carer report; medical assessment data: sleep disturbance no/yes | From depression questionnaire: sleep disturbance no/yes | **sleep disturbance**  0=no;  1=yes |
| 1. Mobility (impairment) | self-report: no/yes  family report: no/yes | self/family/carer report or medical assessment data: impairment no/yes | self-report: impairment no/yes | **mobility** is**sue**  0=no;  1=yes |
| 1. Falls | self-report: no/yes;  EFST: no falls/1 fall ≥ 2 falls | self/family/carer report or medical assessment data: no/yes;  number of falls -1/2/3/more | self-report: no/yes  EFST: no falls/1 fall ≥ 2 falls | **falls**  0=no;  1= 1 fall;  2= 2 or more falls  **fall**  0=no;  1=yes |
| 1. Incontinence | Self-report: no/yes; IC1 of ICQ no/yes: no/yes;  mmex_incontinence: no/yes | ADL incontinence (bladder/bowel/both) yes/no | IC1 of ICQ: no/yes | **urinary incontinence**  0=no;  1=yes |
| 1. Polypharmacy | medications*  number of drugs (continuous) | medications*  ≥ 5 prescribed medications | medications*  ≥ 5 prescribed medications | **polypharmacy**  ≥ 5 prescribed medications  0=no;  1=yes |
| 1. Medications | medications list | medications list | medications list | **medications by categories** anticholinergic; antipsychotics; analgesics; anti-epileptic; antithrombotic; diabetic medication; antihypertensive; lipid lowering medications; benzodiazepines and other sedatives  0=no;  1=yes |
| **OUTCOME MEASURES** | | | | |
| 1. Dementia screening tools | KICA-Cog | mKICA | KICA-Cog | **KICA scores 1-16**  **KICA-total**  Scoring for each of the 16 items and total score.  KICA-Cog or mKICA score categorised >36 = 0; ≤35 = 1 |
| 1. Activities of Daily Living | KICA-ADL  Function questions, & service utilisation and informant history | KDS of 8 items  ADL score based on all items dichotomised into nil vs. any impairment | KICA-ADL  Function questions, informant history & service utilisation and informant history | **ADLCook**  **ADLHouse**  **ADLDress**  0=independent; 1=assistance required; 2=dependent  **Any ADL** impairment  0=no;  1=yes |
| 1. Dementia diagnoses & subtyping | DSM-IV  ICD-10 | DSM-IV  National Institute on Aging and Alzheimer’s Association (NIA-AA) | DSM-IV | **Diagnosis#**  0=normal;  1=CIND;  2= Dementia  Dementia type:  DSM-IV classifications: dementia not otherwise specified; dementia of the Alzheimer’s type; vascular dementia, dementia due to other general medical condition, dementia due to multiple aetiologies |

*ADL= Activities of Daily Living; AUDIT-C =Alcohol use disorders identificantion test for consumption; BMI=Body Mass Index; DSM-IV=Diagnostic and Statistical Manual-IV; EFST: Elderly Falls Screening Test; KICA= Kimberley Indigenous Cognitive Assessment; mKICA= modified Kimberley Indigenous Cognitive Assessment; KICA-ADL= Kimberley Indigenous Cognitive Assessment-Activities of Daily Living; KICA-Cog=Kimberley Indigenous Cognitive Assessment-Cognitive; KICA-Dep= Kimberley Indigenous Cognitive Assessment- Depression Scale; KDS=Kilsyth Disability Scale; MMSE= Mini-Mental State Examination;*

**mmex refers to medical history from primary care software*

*~drug_(condition name) refers to medication history from primary care software*

***#****Diagnosis of normal, CIND and Dementia are re-classifications from DSM-IV diagnoses*

**Appendix 1.2: Harmonised longitudinal dataset and variables of interest**

|  | **KHAP W2** | **KGOWS** | **COMBINED** |
| --- | --- | --- | --- |
| 1. Age (years) | Continuous | Continuous | **age**  continuous |
| 1. Sex | Female/male | Female/male | **sex**  0=female;  1=male |
| 1. Locality | Kimberley (all remote) | Urban/regional | **locality**  1=urban;  2=regional;  3=remote |
| 1. Education attainment | Self-reports: some schooling yes/no | Years continuous | **education**  0=no school;  1=some school |
| 1. Smoking status | Self-reports: Current smoking no/yes  Previous smoking no/yes | Self-reports: Current smoking no/yes  Previous smoking no/yes | **smoking**  0= never smoked;  1= ex-smoker;  2=current smoker |
| 1. Alcohol | Self-reports: (alcohol status) Alcohol no/yes  Drink when younger no/yes  (current alcohol intensity) Drank most days/got drunk  Amount: how often get drink only sometimes vs.1-3 days/wk vs. 4-6 days/week vs. every day  (past alcohol intensity)  Age started drinking: 4 categorical age brackets  Age started: as a kid (1); 18-25 yrs (2); >10 years ago (3); 2-10 years ago (4) NA (6)  Drink most days when younger yes(1)/no(0)/NA(2)  Get drunk when younger yes(1)/no(0)/AN(2)  How often get drunk when younger: sometimes (1); 1-3 days per week (2); 4-6 days per week (3); everyday (4); NA(5)  Ceased (1-7) | AUDIT-C: Current alcohol Abstinent/low risk/ high risk;  Past alcohol Abstinent/low risk/ high risk | **alcohol**  0= no alcohol;  1= ex-alcohol;  2= current alcohol  **alcohol current intensity**  0=abstinent  1=low risk/only sometimes/1-3 days/wk/4-6 days/wk and 1-3 drinks;  2=high risk/everyday/4-6 drinks and 4-6 drinks a day/until drunk  **alcohol past intensity**  0=abstinent;  1=low risk/only sometimes/1-3 days/wk/4-6 days/wk and 1-3 drinks;  2=high risk/everyday/4-6 drinks and 4-6 drinks a day/until drunk |
| 1. Diabetes | self-report: no/yes  family report: no/yes | self/family/carer report or medical assessment data: no/yes  medications* | **diabetes**  0=no;  1=yes |
| 1. Hypertension | self-report: no/yes  family report: no/yes | self/family/carer report or medical assessment data: no/yes  SBP/DBP/MAP | **hypertension**  0=no;  1=yes |
| 1. Renal disease | self-report: no/yes  family report: no/yes | self/family/carer report or medical assessment data: no/yes | **renal**  0=no;  1=yes |
| 1. Cardiovascular disease | self-report: no/yes  family report: no/yes | self/family/carer report or medical assessment data: no/yes | **cardiac**  0=no;  1=yes |
| 1. Cerebral vascular disease/stroke | self-report: no/yes  family report: no/yes | self/family/carer report or medical assessment data: no/yes | **stroke**  0=no;  1=yes |
| 1. Hearing problems | self-report: ears good no/yes/don’t know (NB. item reversed) | self/family/carer report or medical assessment data: no/mild vs. moderate/severe | **vision**  0=No/mild/don’t know;  1=moderate/severe |
| 1. Vision problems | self-report: eyes good no/yes/don’t know (NB. item reversed) | self/family/carer report or medical assessment data: no/mild vs. moderate/severe | **hearing**  0=No/mild/don’t know;  1=moderate/severe |
| 1. Head injury with loss of consciousness | self-report: no/yes  family report: no/yes | self/family/carer report or medical assessment data: no/yes | **head injury**  0=no;  1=yes |
| 1. Depression | self-report: Feel down, sad or no good all the time: no/yes  family report: sad all the time no/yes;  antidepressant no/yes | self/family/carer report or medical assessment data: history of depression no/yes  mPHQ9 score (continuous) | **depression**  0=mPHQ9 < 9 or no on personal or informant history;  1=mPHQ9 ≥ 9 or yes on personal or informant history |
| 1. Sleep disturbance | From depression questionnaire: sleeping not enough or too much no/yes | From depression questionnaire; self/family/carer report; medical assessment data: sleep disturbance no/yes | **sleep disturbance**  0=no;  1=yes |
| 1. Mobility (impairment) | self-report: no/yes  family report: no/yes | self/family/carer report or medical assessment data: impairment no/yes | **mobility** is**sue**  0=no;  1=yes |
| 1. Fall/s | self-report: no/yes;  family report: no/yes | self/family/carer report or medical assessment data: no/yes;  number of falls -1/2/3/more | **falls**  0=no;  1= 1 fall;  2= 2 or more falls  **fall**  0=no;  1=yes |
| 1. Incontinence | Self-report: no/yes;  family report: no/yes | ADL incontinence (bladder/bowel/both) yes/no | **urinary incontinence**  0=no;  1=yes |
| 1. Polypharmacy | number of drugs (continuous) | medications list  ≥ 5 prescribed medications | **polypharmacy**  ≥ 5 prescribed medications  0=no;  1=yes |
| ***OUTCOME MEASURES*** | | | |
| 1. Dementia screening tools | KICA-Cog | mKICA | **KICA scores 1-16**  **KICA-total**  Scoring for each of the 16 items and total score.  KICA-Cog or mKICA score categorised >36 = 0; ≤35 = 1 |
| 1. Activities of Daily Living | Family report of ADLs - cooking, cleaning, dressing, showering, medication management, handling money, work, activities and hobbies, driving | KDS of 8 items  ADL score based on all items dichotomised into no trounle vs. any impairment | **ADLCook**  **ADLHouse**  **ADLDress**  0=independent/no trouble  1=any impairment/assistance required |
| 1. Dementia diagnoses & subtyping Diagnosis | DSMI-IV | DSM-IV | **Diagnosis#**  0=normal;  1=CIND;  2= Dementia  Dementia type:  DSM-IV classifications: dementia not otherwise specified; dementia of the Alzheimer’s type; vascular dementia, dementia due to other general medical condition, dementia due to multiple aetiologies |

*ADL= Activities of Daily Living; DSM-IV=Diagnostic and Statistical Manual-IV; EFST: Elderly Falls Screening Test; KICA= Kimberley Indigenous Cognitive Assessment; mKICA= modified Kimberley Indigenous Cognitive Assessment; KICA-ADL= Kimberley Indigenous Cognitive Assessment-Activities of Daily Living; KICA-Cog=Kimberley Indigenous Cognitive Assessment-Cognitive; KICA-Dep= Kimberley Indigenous Cognitive Assessment- Depression Scale; MMSE= Mini-Mental State Examination;*

***#****Diagnosis of normal, CIND and Dementia are re-classifications from DSM-IV diagnoses*
